# Integrated multi-omics profiling reveals novel molecular biomarkers and pathways associated with Fragile X-associated tremor/ataxia syndrome

**DOI:** 10.3389/fnmol.2026.1752903

**Published:** 2026-04-17

**Authors:** Marwa Zafarullah, Matthew Ponzini, Kyoungmi Kim, Paul J. Hagerman, Randi J. Hagerman, Flora Tassone

**Affiliations:** 1 Department of Biochemistry and Molecular Medicine, University of California, Davis, Sacramento, CA, United States; 2 Department of Neurology and Neurological Sciences, Stanford School of Medicine, Stanford, CA, United States; 3 Department of Public Health Sciences, School of Medicine, University of California, Davis, Davis, CA, United States; 4 MIND Institute, University of California, Davis, Sacramento, CA, United States; 5 Department of Pediatrics, University of California, Davis, Sacramento, CA, United States

**Keywords:** amine, biomarkers, Fragile X- associated tremor/ataxia syndrome, FXTAS, lipidomic, metabolomics, multi-omics, premutation

## Abstract

**Introduction:**

Fragile X-associated tremor/ataxia syndrome (FXTAS) is a late-onset neurodegenerative disorder affecting carriers of premutation expansions (55–200 CGG repeats) in the fragile X messenger ribonucleoprotein 1 (*FMR1*) gene. Despite its clinical significance, FXTAS currently lacks reliable molecular markers for disease monitoring and evaluation of therapeutic efficacy.

**Methods:**

To address this critical gap, we performed an integrated multi-omics study combining plasma metabolomics (lipidomics, amine, and primary metabolites) with proteomics analyses in plasma and peripheral blood mononuclear cells (PBMCs) from FXTAS participants (*n* = 5, FXTAS stages 3–5) and age-matched non-carrier healthy controls (HC, *n* = 15).

**Results:**

Integrated analyses revealed molecular differences distinguishing FXTAS from HC, including alterations in metabolites related to energy metabolism (e.g., UDP-glucuronic acid, succinic acid, mannose), lipids (e.g., cholesterol, triglycerides, glycerophospholipids, ceramide), and selected amines (e.g., cystine, glycerophosphocholine, histidine). Proteomic analyses identified proteins associated with FXTAS clinical stage and CGG repeat size, implicating pathways related to mitochondrial function, immune-inflammatory signaling, and lipid metabolism. Comparative analysis of plasma and PBMC proteomes identified Basigin (CD147) and phospholipid transfer protein C2CD2 as overlapping candidate markers across biological matrices.

**Discussion:**

Although limited by sample size and the cross-sectional design, this exploratory study demonstrates the value of integrated, cross-matrix multi-omics profiling for identifying molecular patterns associated with advanced FXTAS. These findings reinforce prior mechanistic models and provide a foundation for future validation in larger, longitudinal cohorts.

## 1 Introduction

Neurodegenerative disorders represent a heterogeneous group of progressive neurological conditions characterized by selective neuronal vulnerability, leading to motor, cognitive, and behavioral impairments (Dugger and Dickson, 2017; Gitler et al., 2017). Fragile X-associated tremor/ataxia syndrome (FXTAS) is a late-onset neurodegenerative disorder primarily affecting older individuals (>50 years) and caused by a repeat expansion (55–200 CGG) located in the 5’UTR of the Fragile X Messenger Ribonucleoprotein 1 (FMR1) gene (Jacquemont et al., 2003; Leehey et al., 2008; Hagerman and Hagerman, 2016, 2021). The prevalence of FXTAS in the general population is estimated to be approximately 1 in 4,000 males and 1 in 7,800 females, varying with the study populations and applied diagnostic criteria (Jacquemont et al., 2004; Rodriguez-Revenga et al., 2009). Clinically, FXTAS is characterized by progressive intention tremor, cerebellar gait ataxia, parkinsonism, cognitive decline, peripheral neuropathy, autonomic dysfunction, and psychiatric disturbances, with significant variability in symptom severity and progression among affected individuals (Berry-Kravis et al., 2007; Apartis et al., 2012; Hall et al., 2014). Neuropathologically, FXTAS is distinguished by the presence of eosinophilic, ubiquitin-positive intranuclear inclusions within neurons and astrocytes, predominantly localized in the cerebellum, brainstem, and cerebral white matter (Greco et al., 2006; Louis et al., 2006; Tassone et al., 2012; Ariza et al., 2016). The underlying molecular mechanism involves RNA-mediated toxicity, resulting from elevated levels of expanded CGG-repeat-containing FMR1 mRNA (Tassone et al., 2000), which sequesters critical RNA-binding proteins and disrupts cellular homeostasis (Jin et al., 2007; Sellier et al., 2010; Todd and Paulson, 2010). Additional molecular mechanisms that may play a role in the pathogenesis of FXTAS include co-transcriptional R-loop formations and the repeat-associated non-AUG (RAN)-initiated translation of potentially toxic proteins, reviewed in Tassone et al. (2023). Currently, there are no targeted therapies or reliable biomarkers available for FXTAS, underscoring the urgent need for research aimed at identifying promising candidate biomarkers that lead to the development of effective treatments.

Multi-omics approaches integrate data from multiple biological disciplines, including genomics, transcriptomics, proteomics, metabolomics, and epigenomics, to provide comprehensive insights into disease mechanisms and identify potential biomarkers and therapeutic targets (Hasin et al., 2017; Subramanian et al., 2020). In recent years, multi-omics studies have significantly advanced our understanding of various neurodegenerative disorders, including Alzheimer’s disease (AD) (Lin et al., 2019; Bai et al., 2020; Johnson et al., 2020; Kodam et al., 2023; Reddy et al., 2024), Parkinson’s disease (PD) (Havelund et al., 2017; Shao and Le, 2019; Tolosa et al., 2020; Schilder et al., 2022; Cannas et al., 2025), amyotrophic lateral sclerosis (ALS) (Umoh et al., 2018; Joilin et al., 2019; Baxi et al., 2022), Huntington’s disease (HD) (Graham et al., 2016; Langfelder et al., 2016; Skene et al., 2018; Wertz et al., 2020), and frontotemporal dementia (FTD) (Swarup et al., 2019; Menden et al., 2023). These studies have uncovered key molecular alterations, dysregulated pathways, and altered protein networks underlying these neurodegenerative diseases, thereby facilitating the discovery of novel and reliable potential biomarkers and ongoing investigation as potential therapeutic targets. Proteomic analyses conducted on human postmortem FXTAS brain tissues have revealed significant alterations in proteins involved in mitochondrial dysfunction, oxidative stress, RNA metabolism, and neuroinflammation, highlighting potential pathogenic pathways underlying FXTAS neuropathology (Holm et al., 2021; Abbasi et al., 2022; Yao et al., 2024). Additionally, metabolomic studies performed on FXTAS brain (Salcedo-Arellano et al., 2023), and plasma samples from FXTAS participants have identified distinct metabolic signatures, including dysregulated energy metabolism, altered amino acid profiles, and perturbed lipid metabolism, suggesting systemic metabolic disturbances associated with the disease (Giulivi et al., 2016a,b; Kong et al., 2019). In these studies, the lipidomic analyses have further indicated significant alterations in lipid species, particularly phospholipids and sphingolipids, in both plasma and fibroblasts derived from FXTAS participants, implicating lipid dysregulation as a potential contributor to FXTAS pathogenesis (Giulivi et al., 2016a,b; Song et al., 2016; Kong et al., 2019). Furthermore, the proteomic and metabolomic studies conducted on peripheral blood mononuclear cells (PBMCs) from FXTAS participants have identified altered expression of proteins and metabolites involved in mitochondrial bioenergetics, oxidative stress response, and immune regulation, providing additional evidence of systemic molecular alterations in FXTAS (Napoli et al., 2016). Recent longitudinal multi-omics studies have focused on identifying molecular signatures and candidate biomarkers associated with the diagnosis and progression of FXTAS in individuals who developed the disorder over time. In this context, we conducted a series of integrative omics analyses, including metabolomics and proteomics, in carriers of a premutation allele who were followed longitudinally over several years (Zafarullah et al., 2020, 2021, 2023) with and without symptoms, and identified significant dysregulation in lipid metabolism. Specifically, metabolites in the glycerophospholipids and sphingolipids pathways were altered only in those with the FXTAS diagnosis, highlighting lipid dysregulation as a key pathological feature of the disorder and suggesting these metabolites as potential biomarkers for FXTAS progression (Zafarullah et al., 2020). Later, in a subsequent study we analyzed the plasma metabolomics data with structural MRI imaging, revealing significant correlations between specific metabolite profiles and brain measures (Zafarullah et al., 2021), suggesting their potential use as early biomarkers for structural brain changes and FXTAS risk assessment in premutation carriers. Lastly, in a most recent study, we performed proteomic analyses on blood samples from premutation carriers, identifying altered expression of proteins involved in inflammation, mitochondrial dysfunction, oxidative stress, and neurodegeneration pathways in agreement with previous studies (Holm et al., 2021; Abbasi et al., 2022; Yao et al., 2024). These proteomic alterations further support the involvement of these pathways in FXTAS pathogenesis and provide potential biomarkers for early detection and therapeutic intervention (Zafarullah et al., 2023).

Prior studies have largely focused on a single molecular layer or one biological matrix, which may overlook shared molecular signatures across systemic circulation and cellular compartments. As in our previous studies, we only focused on individuals who developed FXTAS over time and were at FXTAS stages 2 or 3 of the disorder. However, in the current study, we present an integrated multi-omics investigation combining metabolomics and proteomics analyses in individuals with advanced stages of FXTAS (*n* = 15) compared to non-carrier HC (*n* = 15), aimed at validating previous findings, better understanding the progression of the disorder, and identifying key markers associated with disease advancement. Additionally, plasma and PBMCs are accessible, minimally invasive biological samples, making them ideal for biomarker discovery and disease monitoring in neurological disorders. We performed proteomic profiling in both plasma and PBMCs, and metabolomic profiling in plasma samples derived from the same individuals. Integrating both metabolomics and proteomics data from the same subjects provides a unique opportunity to uncover coordinated molecular alterations and pathway-level interactions that may not be clear when each omics approach is analyzed independently. We confirm the recurrent involvement of previously reported metabolic and proteomic pathways, in FXTAS progression, and uncovered additional molecular features that may warrant further investigation as candidate biomarkers. Importantly, the simultaneous integration of plasma and PBMC proteomes with plasma metabolomics in individuals with advanced FXTAS enables cross-matrix comparisons that are not achievable in single-omics or single-tissue studies, providing insight into coordinated systemic and cellular molecular alterations.

## 2 Materials and methods

### 2.1 Study participants

We characterized a comprehensive multi-omics profiling, including metabolomics and proteomics, on 15 male participants with FXTAS, over the age of 45 years, and 15 male participants non-carrier age-matched HC. The studies and all protocols were carried out in accordance with the Institutional Review Board at the University of California, Davis. All participants gave written informed consent before participating in the study in line with the Declaration of Helsinki. FXTAS scoring was based on the clinical assessment as previously described (Bacalman et al., 2006), and three diagnosis categories, termed “definite,” “probable,” and “possible” FXTAS, were used for the diagnosis of FXTAS as previously described (Jacquemont et al., 2003; Hall et al., 2014).

### 2.2 CGG repeat sizing

Genomic DNA (gDNA) was isolated from 5 mL of peripheral blood leukocytes in EDTA-containing blood collection tubes using the Gentra Puregene Blood Kit (Qiagen). CGG repeat allele sizing and methylation status were assessed by using a combination of Southern blot and PCR analysis, an Alpha Innotech FluorChem 8800 Image Detection System, and the ABI 3730XL 96-Capillary Electrophoresis Genetic Analyzer (Applied Biosystems). Details of the protocols are as previously reported (Tassone et al., 2008; Filipovic-Sadic et al., 2010).

### 2.3 Sample handling and preparation

Peripheral blood was collected in BD Vacutainer™ CPT™ vacutainers with sodium citrate (Becton Dickinson, United States) and centrifuged according to the manufacturer’s recommendations for separating mononuclear cells from whole blood. PBMCs were washed with Dulbecco’s phosphate-buffered saline (PBS), frozen in RPMI 1640 media with 10% fetal bovine serum and 10% dimethyl sulfoxide and stored in liquid nitrogen until use. For plasma isolation, the blood samples were collected into EDTA-containing blood collection tubes, centrifuged for 10 min at 1,000 × *g*, and the isolated plasma was stored at −80 °C until further processing.

#### 2.3.1 Sample preparation for metabolomics profiling

All solvents used were of HPLC grade or higher (Fisher Scientific, Waltham, MA, United States). Internal standards (D3 decanoyl-L-carnitine, D3 dodecanoyl-L-carnitine, D3 octadecanoyl-L-carnitine, D3 palmitic acid, D9 oleic acid, D11 arachidonic acid, D7 cholesterol, C17 sphingosine, Ultimate SplashOne) were purchased from various suppliers (Cambridge Isotopes, Avanti Polar Lipids). Blood plasma samples were prepared as follows: a cold extraction solvent of methanol with internal standards was added to 20 μL of thawed plasma aliquots. MTBE was then added, followed by shaking and the addition of LC/MS grade water. Centrifugation separated the mixture into two layers. The upper (non-polar) layer was used for lipidomics LC-MS analysis using reverse-phase chromatography, while the lower (polar) layer was used for LC-MS metabolomics using HILIC chromatography and for GC-MS analysis. Portions of each phase were dried and stored at −20 °C until analysis. Prior to analysis, the dried samples were reconstituted and derivatized, if needed, using the appropriate solvent and internal standards mix. For quality control, a method blank (water) and pooled samples were processed alongside the experimental samples for every 10 samples.

#### 2.3.2 Sample preparation for proteomic profiling

Frozen, isolated PBMCs were quickly thawed in a 37 °C water bath and transferred into a 1.5 mL tube and spun for 20 min to pellet the cells. The freezing media was removed, and proteins were extracted in 50 mM Triethyl Ammonium Bicarbonate (TEAB) with 5% SDS. Protein concentration was determined by Pierce BCA assay (ThermoFisher, United States). A total of 150 μg of proteins was digested on an S-Trap™ Digestion column plate. After reduction and alkylation (DTT then IAA), the samples were acidified with 12% phosphoric acid, followed by the addition of freshly made S-trap buffer. The entire acidified lysate was transferred to the S-trap plate and pushed through. After washing, trypsin digestion was performed overnight at 37 °C. Peptides were then eluted and dried. Peptides were resuspended in 0.1% TFA 2% ACN and quantified using Pierce™ Quantitative Fluorometric Peptide Assay (Thermo Fisher Scientific). Abundant plasma proteins were depleted using a perchloric acid precipitation method as detailed in Viode et al. (2023). Briefly, 50 μL of plasma was diluted to 450 μL of water, and 25 μL of perchloric acid was added. After vigorous agitation and freezing, supernatants were retained and cleaned with a uSPE HLB plate. Proteins were eluted, dried, resuspended in 50 mM ammonium bicarbonate, and digested with trypsin overnight. The digestion was stopped with 5 μL of 10% formic acid and analyzed by LC-MS/MS.

### 2.4 Liquid chromatography, gas chromatography, and mass spectrometry techniques

For the metabolomics, lipidomics, and amines analyses, data were acquired using liquid chromatography high-resolution mass spectrometry (LC-HRMS) and gas chromatography mass spectrometry (GC-MS) instruments.

For the proteomics, each sample (500 ng total peptide) was loaded onto a disposable Evotip C18 trap column (Evosep Biosystems, Denmark). NanoLC was performed on an Evosep One instrument (Evosep Biosystems), eluting directly onto a PepSep analytical column. Mobile phases A and B were water with 0.1% formic acid and 80:20 acetonitrile:water with 0.1% formic acid, respectively. The standard pre-set method of 100–60–30 samples-per-day was used (11.5–21–44 min runs). Mass spectrometry was performed on a hybrid trapped ion mobility spectrometry-quadrupole time of flight mass spectrometer (timsTOF Pro, Bruker Daltonics), operated in PASEF mode. De-solvated ions entered the vacuum region, and DIA was performed with appropriate precursor windows. Data were processed as described below.

### 2.5 Data analysis

For metabolomics, data were processed according to standard operating protocols (West Coast Metabolomics Center). For proteomics, DIA data were processed using Spectronaut 18.5 (Biognosys). A direct DIA workflow with default settings was used. Search included trypsin with two missed cleavages, carbamidomethyl as fixed modification, and acetyl (protein N-term) plus oxidation as variable modifications. Statistical analysis was conducted using the R package (R 4.2.3 or 4.3.1). Data were quantity-normalized, log_2_-transformed, and imputed for missing values using half-minimum imputation prior to statistical analysis. Partial least squares–discriminant analysis (PLS-DA) was used to explore group separation and identify most influential metabolites/lipids/proteins based on variable importance in the projection (VIP) scores. Stability of variable selection of most influential metabolites/lipids/proteins was evaluated using k-fold cross-validation (k = 5). Differential analysis was conducted to identify metabolites/lipids/proteins whose expression differed significantly between groups using Analysis of Covariance (ANCOVA) with age included as a covariate. Linear regression was performed to assess relationships between expression levels and CGG repeat size or FXTAS stage (controlling for age). Multiple testing corrections were applied using the Benjamini-Hochberg false discovery rate (FDR) procedure. Significance was determined at an FDR < 0.1 for PSM and protein group identification.

## 3 Results

### 3.1 Subjects

The mean (SD) age of healthy controls (HC) was 64.22 (5.39) years, ranging from 55.3 to 73.3 years, while in participants with FXTAS, the mean (SD) age was 64.07 (5.42) years, ranging from 53.50 to 72.67 years. The number of CGG repeats in the healthy control (HC) group had a mean (SD) of 27.1 (4.2), ranging from 19 to 34. In contrast, the FXTAS group showed a significantly higher mean (SD) of 95.5 (15.3), with a range of 66 to 133 repeats. Among the FXTAS participants, disease severity varied, with five individuals (33.3%) at stage 3, six individuals (40.0%) at stage 4, and four individuals (26.7%) at stage 5. Participants’ ages did not differ significantly, while CGG repeat numbers were significantly lower in HC than FXTAS participants (*p* < 0.001). Of the FXTAS subjects, five were FXTAS stage 3, six were FXTAS stage 4, and four were FXTAS stage 5.

### 3.2 Primary metabolite profiling of HC and FXTAS participants

**FIGURE 1:**
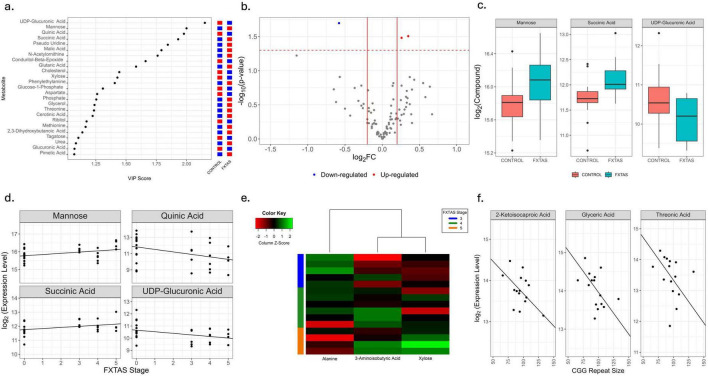
Comparative plasma primary metabolite profile between HC and FXTAS patients. **(a)** Variable importance in the projection (VIP) score plot for the top 25 most influential metabolites including glutaric acid (PC 16:0_glutaric acid). Red represents up-regulation and blue represents down-regulation. **(b)** Volcano plot showing fold changes and *p*-values of significance of metabolites between control and FXTAS samples. Log2 of the fold change is represented on the x-axis and –log10 of the *p*-value on the y-axis. The significant metabolites are colored. Red represents up-regulation in FXTAS samples and blue represents down-regulation in FXTAS samples as compared to HC. **(c)** Box plots showing the distribution of significantly differentially expressed metabolites between HC and FXTAS patients at *p* < 0.05. The heavy line in each box represents the median, the lower and upper box edges represent the 25th and 75th percentiles, respectively, and the lower and upper whiskers represent the smallest and largest observations, respectively. **(d)** Scatterplots with fitted regression lines of FXTAS stage against expression of metabolites significantly associated with FXTAS stage at *p*-value < 0.05. **(e)** Heatmap of metabolites that are significantly associated with FXTAS stage in FXTAS patients at *p* < 0.05. **(f)** Scatterplots with fitted regression lines of CGG repeat size against expression of metabolites significantly associated with CGG repeat number in all samples at FDR < 0.1.

A PLS analysis showed that the untargeted plasma metabolomics profiling could segregate FXTAS (*n* = 15) from HC (*n* = 15) (Supplementary Figure 1a). The first two PLS components explained 81.06% of the variance (Supplementary Figure 1b). The 25 most influential metabolites in PLS segregation are shown in Figure 1a. Among 103 metabolites (Supplementary Table 1), three were significantly differentially expressed (*p* < 0.05, Figure 1b), including downregulation of UDP-glucuronic acid and upregulation of succinic acid and mannose, in FXTAS compared to HC (Figure 1c). Additionally, four metabolites (mannose, UDP-glucuronic acid, quinic acid, and succinic acid) suggested significant associations with FXTAS stage across all samples (*n* = 30) (*p* < 0.05, Figure 1d). Upon stratification by disease severity, correlation analysis in advanced FXTAS stages (Jacquemont et al., 2003; Leehey et al., 2008; Hagerman and Hagerman, 2016) revealed that 3-aminoisobutyric acid, alanine, and xylose also showed significant associations with disease progression (*p* < 0.05, Figure 1e). Several metabolites (glyceric acid, threonic acid, and 2-ketoisocaproic acid) showed a significant negative correlation of their expression level with CGG repeat number (*p* < 0.05, Figure 1f), but none remained significant at FDR < 0.1.

### 3.3 Lipidomic profiling of HC and FXTAS participants

**FIGURE 2:**
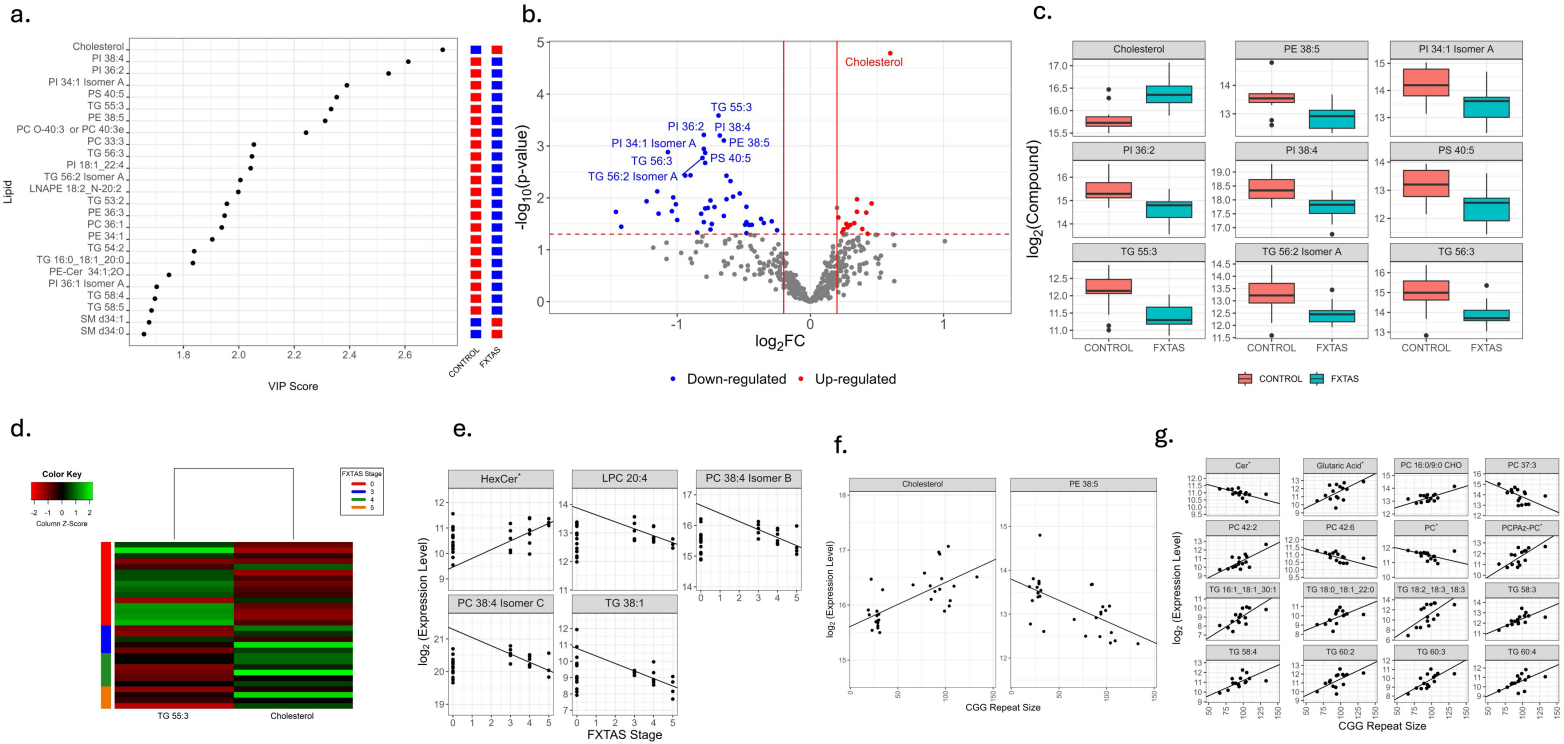
Comparative plasma lipidomic profiling of HC and FXTAS patients. **(a)** Variable importance in the projection (VIP) score plot for the top 25 most influential lipids. Red represents up-regulation and blue represents down-regulation. **(b)** Volcano plot showing fold changes and *p*-values of significance of lipids between control and FXTAS samples. Log2 of the fold change is represented on the x-axis and –log10 of the *p*-value on the y-axis. The significant lipids are colored. Red represents up-regulation in FXTAS samples and blue represents down-regulation in FXTAS samples as compared to control samples. Lipids significant at FDR < 0.1 are labeled. **(c)** Box plots showing the distribution of significantly differentially expressed lipids between HC and FXTAS patients at FDR < 0.1. The heavy line in each box represents the median, the lower and upper box edges represent the 25th and 75th percentiles, respectively, and the lower and upper whiskers represent the smallest and largest observations, respectively. **(d)** Heatmap of lipids that are significantly associated with FXTAS stage among all samples at FDR < 0.1. **(e)** Scatterplots with fitted regression lines of FXTAS stage against expression of lipids including HexCer (HexCer 34:1;O2| HexCer 18:1;O2/16:0) significantly associated with FXTAS stage at *p*-value < 0.05. **(f)** Scatterplots with fitted regression lines of CGG repeat size against expression of lipids significantly associated with CGG repeat number in all samples at FsDR < 0.1. **(g)** Scatterplots with fitted regression lines of CGG repeat size against expression of lipids including PCPAz-PC (PC 16:0_azealic acid); Cer (Cer 41:2;O2| Cer 18:2;O2/23:0); and PC (PC 42:9| PC 20:4_22:5) significantly associated with CGG repeat number in a subset of samples with FXTAS advance stages at *p* < 0.05.

The 25 most influential lipids in PLS analysis are depicted in Figure 2a. Among 510 lipids analyzed, 60 showed nominal significance (*p* < 0.05; Supplementary Table 2), of which nine survived FDR correction at q < 0.05 and nine at q < 0.1 (Figures 2b, c). Notably, only cholesterol was upregulated, whereas the remaining eight lipids were downregulated in FXTAS. Moreover, 60 lipids exhibited significant associations with FXTAS stage across all samples (*n* = 30) (*p* < 0.05, Supplementary Table 3), with two [cholesterol and triacylglycerides (TG) 55:3] also significant at FDR < 0.1 (Figure 2d). When focusing on advanced FXTAS stages, five additional lipids (PC 38:4 isomer C, TG 38:1, PC 38:4 isomer B, LPC 20:4, and HexCer 34:1;O2) were significantly associated with disease progression (*p* < 0.05, Figure 2e), but none remained significant at FDR < 0.1. Regarding correlations with CGG repeat size, 52 lipids were significant (*p* < 0.05, Supplementary Table 4), with cholesterol and PE 38:5 also significant at FDR < 0.1 (Figure 2f). Among advanced-stage FXTAS participants, 16 lipids were significantly associated with CGG repeat size (*p* < 0.05, Figure 2g) but did not meet FDR < 0.1.

### 3.4 Amine profiling of HC and FXTAS participants

**FIGURE 3:**
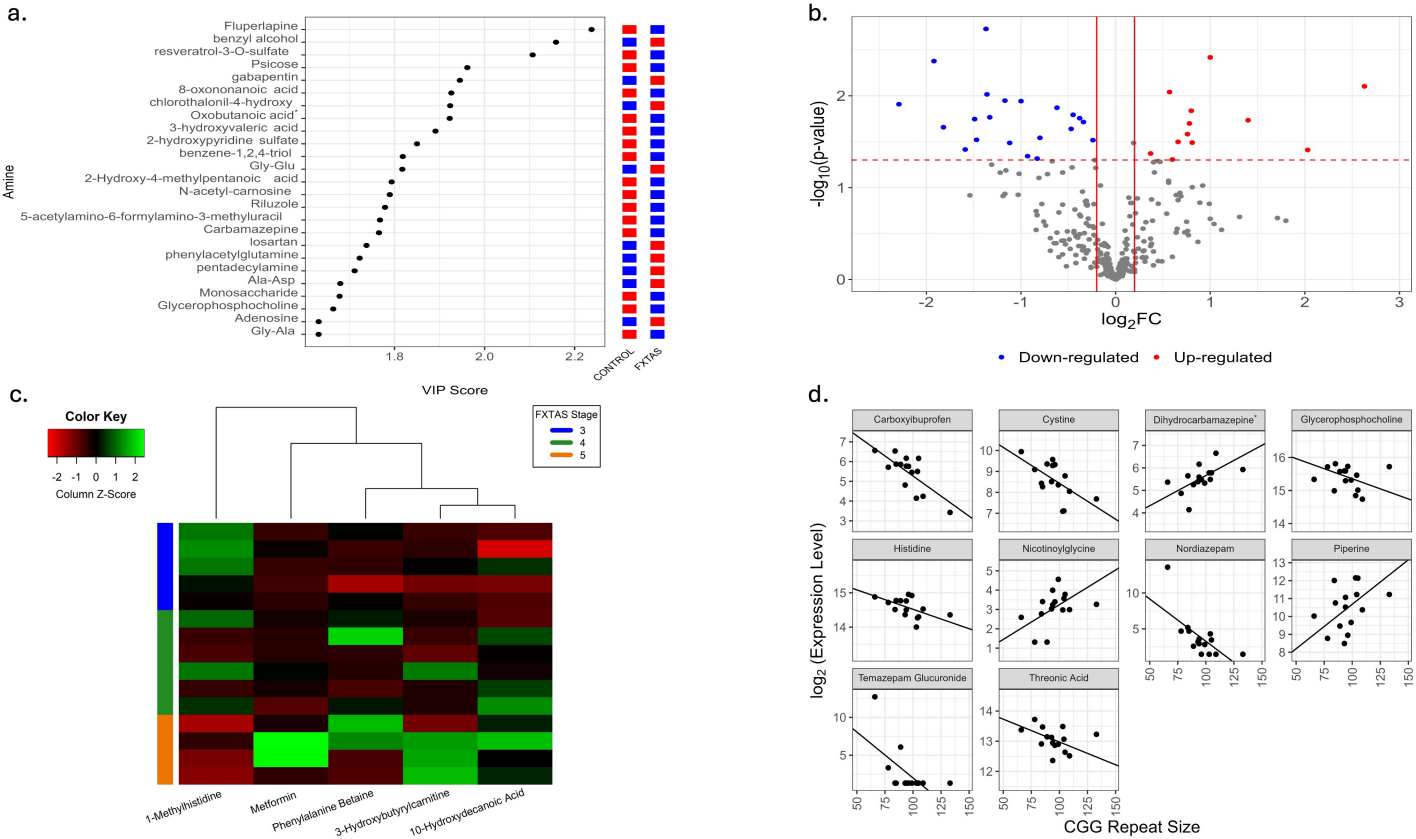
Comparative plasma amine profile analysis between HC and FXTAS patients. **(a)** Variable importance in the projection (VIP) score plot for the top 25 most influential amines. Red represents up-regulation and blue represents down-regulation. **(b)** Volcano plot showing fold changes and *p*-values of significance of amines between control and FXTAS samples. Log2 of the fold change is represented on the x-axis and –log10 of the *p*-value on the y-axis. The significant amines are colored. Red represents up-regulation in FXTAS samples and blue represents down-regulation in FXTAS samples as compared to control samples. **(c)** Heatmap of amines that are significantly associated with FXTAS stage in FXTAS patients at *p* < 0.05. **(d)** Scatterplots with fitted regression lines of CGG repeat size against expression of amines including Dihydrocarbamazepine (cis-10,11-Dihydroxy-10,11-dihydrocarbamazepine) significantly associated with number of CGG repeats in subset of samples with FXTAS advance stages at *p* < 0.05.

The 25 most influential amines in PLS analysis are shown in Figure 3a. Among 288 plasma amines, 34 showed significant differential expression between HC and FXTAS (*p* < 0.05, Figure 3b, Supplementary Table 5), although none remained significant at FDR < 0.1. Thirty-five amines were significantly associated with FXTAS stage (*p* < 0.05, Supplementary Table 6), with five of these (1-methylhistidine, 10-hydroxydecanoic acid, 3-hydroxybutyrylcarnitine, phenylalanine betaine, and metformin) achieving *p* < 0.05 but not FDR < 0.1 (Figure 3c). Ten amines were significantly associated with CGG repeat number (*p* < 0.05, Supplementary Table 7 and Figure 3d), none surviving FDR < 0.1. While none of the amines survived FDR correction (*q* < 0.05), nominally significant findings are reported for hypothesis generation and should be interpreted cautiously pending validation in larger cohorts.

### 3.5 Proteomic PBMC profiling in HC and FXTAS participants

**FIGURE 4:**
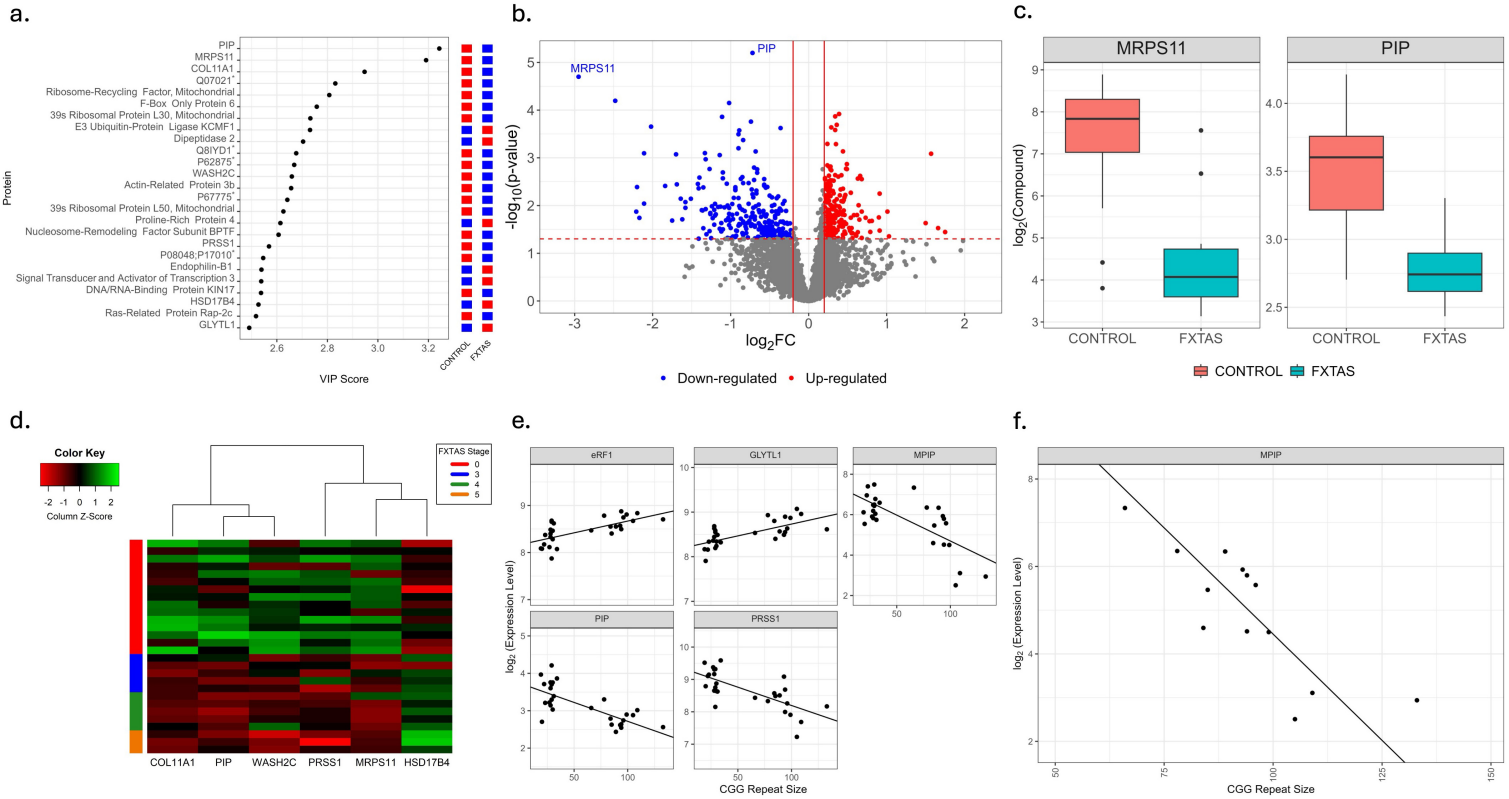
Comparative PBMCs proteomic profiling in HC and FXTAS patients. **(a)** Variable importance in the projection (VIP) score plot for the top 25 most influential proteins in PBMC, including Complement component 1 Q subcomponent-binding protein, mitochondrial (Q07021); eukaryotic peptide chain release factor GTP-binding subunit ER (Q8IYD1); DNA-directed RNA polymerases I, II, and III subunit RPABC5 (P62875); serine/threonine-protein phosphatase 2A catalytic subunit alpha isoform (P67775); and zinc finger Y-chromosomal protein/zinc finger X-chromosomal protein (P08048; P17010). Red represents up-regulation and blue represents down-regulation. **(b)** Volcano plot showing fold changes and *p*-values of significance of PBMC proteins between control and FXTAS samples. Log2 of the fold change is represented on the x-axis and –log10 of the p value on the y-axis. The significant proteins in PBMC are colored. Red represents up-regulation in FXTAS samples and blue represents down-regulation in FXTAS samples as compared to control samples. PBMC proteins significant at FDR < 0.1 are labeled. **(c)** Box plots showing the distribution of significantly differentially expressed proteins between control and FXTAS patients at FDR < 0.1. The heavy line in each box represents the median, the lower and upper box edges represent the 25th and 75th percentiles, respectively, and the lower and upper whiskers represent the smallest and largest observations, respectively. **(d)** Heatmap of proteins that are significantly associated with FXTAS stage among all samples at FDR < 0.1. **(e)** Scatterplots with fitted regression lines of CGG repeat size against expression of proteins significantly associated with number of CGG repeats in all samples at FDR < 0.1. **(f)** Scatterplots with fitted regression lines of CGG repeat size against expression of proteins significantly associated with number of CGG repeats in subset of samples with FXTAS advance stages at FDR < 0.1.

Our extensive proteomic analysis of PBMCs revealed a total of 5,925 proteins, with the top 25 most influential in PLS analysis shown in Figure 4a. Among these, 559 proteins were significantly different between HC and FXTAS (*p* < 0.05, Supplementary Table 8), with two (prolactin-inducible protein and 28S ribosomal protein S11 mitochondrial) surviving FDR < 0.1 and both downregulated in FXTAS (Figures 4b, c). Additionally, 510 proteins were significantly associated with FXTAS stage (*p* < 0.05, Supplementary Table 9), with six surviving FDR < 0.1 (Figure 4d). A total of 804 proteins correlated significantly (*p* < 0.05) with CGG repeat number (Supplementary Table 10), five of which remained significant at FDR < 0.1 (Figure 4e). In advanced-stage FXTAS, 359 proteins were significantly associated with CGG repeat number (*p* < 0.05, Supplementary Table 11), with M-phase-specific PLK1-interacting protein remaining significant at FDR < 0.1 (Figure 4f).

### 3.6 Plasma proteomic profiling of HC and FXTAS participants

**FIGURE 5:**
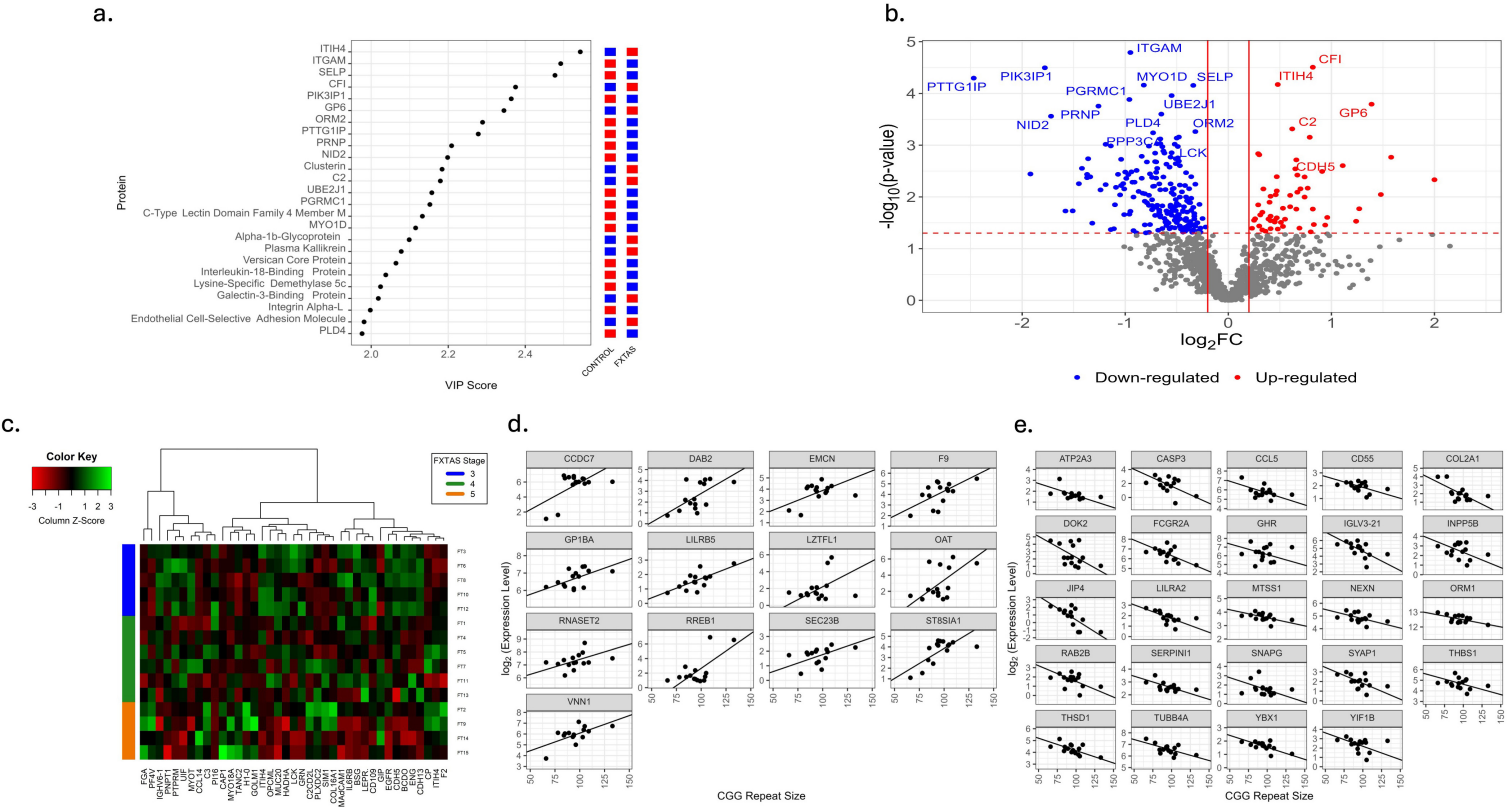
Comparative plasma proteomic profiling of HC and FXTAS patients. **(a)** Variable importance in the projection (VIP) score plot for the top 25 most influential proteins in plasma. Red represents up-regulation and blue represents down-regulation. **(b)** Volcano plot showing fold changes and *p*-values of significance of plasma proteins between HC and FXTAS samples. Log2 of the fold change is represented on the x-axis and -log10 of the p value on the y-axis. The significant plasma proteins are colored. Red represents up-regulation in FXTAS samples and blue represents down-regulation in FXTAS samples as compared to HC. Subset of plasma proteins significant at FDR < 0.1 are labeled. **(c)** Heatmap of proteins significantly associated with FXTAS stage in participants with FXTAS at *P* < 0.05. **(d,e)** Scatterplots with fitted regression lines of CGG repeat number against expression of subset of proteins significantly associated with CGG repeat number in FXTAS samples at *P* < 0.05.

Plasma proteomic analysis revealed 1,348 proteins, with the top 25 most influential in PLS shown in Figure 5a. Among these, 252 proteins were significantly different between HC and FXTAS (*p* < 0.05), with 101 surviving FDR < 0.1 (Figure 5b, Supplementary Table 12). Furthermore, 244 proteins showed significant associations with FXTAS stage (*p* < 0.05), of which 81 remained significant at FDR < 0.1 (Supplementary Table 13). In advanced-stage FXTAS, 39 proteins were significantly associated with disease severity (*p* < 0.05, Figure 5c) but none survived FDR < 0.1. Across all samples, 251 proteins were significantly associated with CGG repeat number (*p* < 0.05), with 85 at FDR < 0.1 (Supplementary Table 14). In advanced-stage FXTAS, 37 proteins correlated with CGG repeat size (*p* < 0.05) but none met FDR < 0.1 (Figures 5d, e).

### 3.7 Comparison of differentially expressed proteins in plasma and PBMCs

We compared PBMC and plasma proteomic profiles between HC and FXTAS and found 252 significantly different proteins in plasma (*p* < 0.05) vs. 559 in PBMCs (*p* < 0.05). Eighteen proteins were significant in both plasma and PBMCs at *p* < 0.05, and none at FDR < 0.1 (Supplementary Figure 2a). For FXTAS stage, 244 proteins showed significant associations in plasma, 513 in PBMCs, with 17 overlapping at *p* < 0.05, none at FDR < 0.1 (Supplementary Figure 2b). In advanced-stage FXTAS, 39 proteins were associated in plasma vs. 192 in PBMCs (*p* < 0.05), with only two (Basigin and phospholipid transfer protein C2CD2) overlapping (Supplementary Figure 2c). For CGG repeat number, 251 proteins were significant in plasma (*p* < 0.05) vs. 810 in PBMCs, with 38 overlapping (none at FDR < 0.1, Supplementary Figure 2d). In advanced-stage FXTAS, 37 were significant in plasma vs. 364 in PBMCs (*p* < 0.05), with three overlapping and none at FDR < 0.1 (Supplementary Figure 2e).

## 4 Discussion

This study uniquely combines metabolomics, lipidomics, amines, and proteomics from both plasma and PBMCs in the same individuals with advanced FXTAS, enabling cross-matrix integration and validation of candidate molecular signatures. The simultaneous plasma-PBMC analysis enabled detection of CD147 and C2CD2 alterations in both matrices, suggesting these proteins reflect both systemic and cellular pathology. Such cross-matrix validation would not be possible in single-matrix studies and substantially increases confidence that these are biologically meaningful changes rather than matrix-specific artifacts. Untargeted plasma metabolomics identified several metabolites significantly altered in FXTAS, highlighting potential metabolic pathways involved in the disease pathogenesis. Notably, UDP-glucuronic acid was downregulated, while succinic acid and mannose were upregulated in FXTAS, consistent with earlier findings (Zafarullah et al., 2020, 2021) suggesting that dysregulated energy and carbohydrate metabolism are hallmarks of FXTAS progression. Elevated succinic acid, a key TCA cycle intermediate, is also linked to reactive oxygen species generation, further implicating mitochondrial dysfunction (Giulivi et al., 2016b; Napoli et al., 2016; Zafarullah et al., 2020). Metabolites such as 3-aminoisobutyric acid and xylose emerged as potentially relevant to advanced stages, validating and expanding on prior work.

Our lipidomic data showed significant alterations in glycerophospholipid species and an upregulation of cholesterol, aligning with previous findings of dysregulated lipid homeostasis (Song et al., 2016; Zafarullah et al., 2020). Cholesterol dysregulation has been implicated in numerous neurodegenerative conditions (Di Paolo and Kim, 2011; Kao et al., 2020). Several phospholipids, triglycerides, and phosphatidylcholines correlated with FXTAS stage or CGG repeat size, especially in participants at advanced stages, suggesting potential roles for these lipids in disease progression. Plasma amine profiling also revealed alterations in amine metabolism in FXTAS (Zafarullah et al., 2020).

Associations with FXTAS stage were noted for 1-methylhistidine, 10-hydroxydecanoic acid, 3-hydroxybutyrylcarnitine, phenylalanine betaine, and metformin, and although none of these associations survived correction for multiple testing, these findings should be viewed as hypothesis-generating and may reflect broader perturbations in amino acid related pathways observed across neurodegenerative disorders. Indeed, the recurring involvement of amino-acid related pathways echo findings in Alzheimer’s and Parkinson’s disease (Havelund et al., 2017; Shao and Le, 2019; Johnson et al., 2020).

By analyzing both plasma and PBMC proteomes, we could probe systemic and cellular potential biomarkers of FXTAS. Notably, prolactin-inducible protein and mitochondrial 28S ribosomal protein S11 were both significantly downregulated in PBMCs at FDR < 0.1, consistent with mitochondrial dysfunction and inflammatory processes previously implicated in FXTAS (Napoli et al., 2016; Zafarullah et al., 2023). Plasma proteomics uncovered numerous proteins associated with disease severity and CGG repeat size, highlighting systemic contributions of immune-inflammatory pathways. Basigin (CD147) and phospholipid transfer protein C2CD2 were identified in both plasma and PBMC proteomes, highlighting coordinated alterations across biological compartments and suggesting potential involvement in lipid dysregulation and immune inflammatory processes (Nahalkova et al., 2010; Zhu et al., 2014). While these overlapping findings are biologically convincing and consistent with prior literature, further validation will be required to assess their relevance as biomarkers or therapeutic targets. Across omics layers and biological matrices, the most consistent associations converged on pathways related to mitochondrial function, lipid metabolism, and immune-inflammatory signaling. In contrast, some metabolite or matrix-specific associations varied more, showing that integrative analyses are needed to distinguish consistent molecular patterns from condition-dependent changes.

However, the modest sample size (*n* = 15 per group) limits statistical power and the generalizability of our findings, particularly for features showing nominal significance without FDR correction. These results should be considered exploratory and hypothesis-generating. The cross-sectional design assessment of potential candidate biomarker trajectories over time or causal inference regarding disease progression. Many nominally significant findings, especially in the amine and FXTAS stage analyses, did not survive multiple testing correction and require validation in larger, independent cohorts. Future studies should include longitudinal follow-up, larger sample sizes, and functional validation experiments to identify causality and clinical utility of identified biomarker candidates.

## 5 Conclusion

In summary, this integrated exploratory multi-omics study identifies combining metabolomics, with plasma and PBMC proteomics identifies candidate molecular signatures associated with late stage FXTAS. Our findings reinforce the involvement of mitochondrial dysfunction, lipid dysregulation, altered amine metabolism, and immune-inflammatory pathways previously implicated in FXTAS and other neurodegenerative disorders. A key strength of this work is the cross-matrix integration of systemic and cellular data from the same individuals, which enables identification of overlapping molecular features including Basigin (CD147) and the phospholipid transfer protein (C2CD2)., This study is exploratory in nature and limited by sample size and cross-sectional design, and several associations did not remain significant after multiple-testing correction. Accordingly, the identified molecular features should be interpreted as candidate signals requiring validation in larger, longitudinal cohorts and functional studies. Future work aimed at validating these findings and elucidating their biological roles will be essential for determining their potential clinical relevance in FXTAS.

## Data Availability

The data supporting the conclusions of this study are available from the corresponding author upon reasonable request.

## Supplementary material

Supplementary Figure 1 The healthy control and Fragile X-associated tremor/ataxia syndrome (FXTAS) groups show high level of segregation. **(a)** Scatter plot showing the separation between control and FXTAS samples. **(b)** Screen plot showing the proportion of variance around 81.06% between both groups.

Supplementary Figure 2 Differentially expressed common proteins identified from PBMC and plasma profiling of healthy control (HC) and Fragile X-associated tremor/ataxia syndrome (FXTAS) patients. Venn diagram of proteins **(a)** significantly differentially expressed (P < 0.05). **(b)** Significantly associated with FXTAS stage at *p* < 0.05 in all samples. **(c)** Significantly associated with FXTAS stage at *p* < 0.05 in FXTAS patients. **(d)** Significantly associated with CGG repeat number at *p* < 0.05 in all samples. **(e)** Significantly associated with CGG repeat number at *p* < 0.05 in FXTAS patients.

## References

[B1] Abbasi D. A. Nguyen T. T. A. Hall D. A. Robertson-Dick E. Berry-Kravis E. Cologna S. M. (2022). Characterization of the cerebrospinal fluid proteome in patients with fragile X-associated tremor/ataxia syndrome. *Cerebellum* 21 86–98. 10.1007/s12311-021-01262-7 34046842

[B2] Apartis E. Blancher A. Meissner W. G. Guyant-Maréchal L. Maltête D. De Broucker T. et al. (2012). FXTAS: new insights and the need for revised diagnostic criteria. *Neurology* 79 1898–1907. 10.1212/WNL.0b013e318271f7ff 23077007

[B3] Ariza J. Rogers H. Monterrubio A. Reyes-Miranda A. Hagerman P. J. Martínez-Cerdeño V. A. (2016). Majority of FXTAS cases present with intranuclear inclusions within purkinje cells. *Cerebellum* 15 546–551. 10.1007/s12311-016-0776-y 27108270

[B4] Bacalman S. Farzin F. Bourgeois J. A. Cogsf J. Goodlin-Jones B. L. Gane L. W. et al. (2006). Psychiatric phenotype of the fragile X-associated tremor/ataxia syndrome (FXTAS) in males: newly described fronto-subcortical dementia. *J. Clin. Psychiatry* 67 87–94. 10.4088/jcp.v67n0112 16426093

[B5] Bai B. Wang X. Li Y. Chen P. C. Yu K. Dey K. K. et al. (2020). Deep multilayer brain proteomics identifies molecular networks in Alzheimer’s disease progression. *Neuron* 105 975–991.e7. 10.1016/j.neuron.2019.12.015. 31926610 PMC7318843

[B6] Baxi E. G. Thompson T. Li J. Kaye J. A. Lim R. G. Wu J. et al. (2022). Answer ALS, a large-scale resource for sporadic and familial ALS combining clinical and multi-omics data from induced pluripotent cell lines. *Nat. Neurosci*. 25 226–237. 10.1038/s41593-021-01006-0 35115730 PMC8825283

[B7] Berry-Kravis E. Abrams L. Coffey S. M. Hall D. A. Greco C. Gane L. W. et al. (2007). Fragile X-associated tremor/ataxia syndrome: clinical features, genetics, and testing guidelines. *Mov. Disord.* 22 2018–2030, quiz 2140. 10.1002/mds.21493. 17618523

[B8] Cannas F. Kopeć K. K. Zuddas N. Cesare Marincola F. Arcara G. Loi M. et al. (2025). Parkinson’s Disease through the lens of metabolomics: a targeted systematic review on human studies (2019-2024). *J. Clin. Med*. 14:6277. 10.3390/jcm14176277 40944038 PMC12429492

[B9] Di Paolo G. Kim T. W. (2011). Linking lipids to Alzheimer’s disease: cholesterol and beyond. *Nat. Rev. Neurosci*. 12 284–296. 10.1038/nrn3012 21448224 PMC3321383

[B10] Dugger B. N. Dickson D. W. (2017). Pathology of neurodegenerative diseases. *Cold Spring Harb. Perspect. Biol*. 9:a028035. 10.1101/cshperspect.a028035 28062563 PMC5495060

[B11] Filipovic-Sadic S. Sah S. Chen L. Krosting J. Sekinger E. Zhang W. et al. (2010). A novel FMR1 PCR method for the routine detection of low abundance expanded alleles and full mutations in fragile X syndrome. *Clin. Chem*. 56 399–408. 10.1373/clinchem.2009.136101 20056738 PMC4031651

[B12] Gitler A. D. Dhillon P. Shorter J. (2017). Neurodegenerative disease: models, mechanisms, and a new hope. *Dis. Model Mech*. 10 499–502. 10.1242/dmm.030205 28468935 PMC5451177

[B13] Giulivi C. Napoli E. Tassone F. Halmai J. Hagerman R. (2016a). Plasma biomarkers for monitoring brain pathophysiology in FMR1 premutation carriers. *Front. Mol. Neurosci*. 9:71. 10.3389/fnmol.2016.00071 27570505 PMC4981605

[B14] Giulivi C. Napoli E. Tassone F. Halmai J. Hagerman R. (2016b). Plasma metabolic profile delineates roles for neurodegeneration, pro-inflammatory damage and mitochondrial dysfunction in the FMR1 premutation. *Biochem. J*. 473 3871–3888. 10.1042/BCJ20160585 27555610 PMC7014977

[B15] Graham S. F. Kumar P. Bahado-Singh R. O. Robinson A. Mann D. Green B. D. (2016). Novel metabolite biomarkers of Huntington’s disease as detected by high-resolution mass spectrometry. *J. Proteome Res*. 15 1592–1601. 10.1021/acs.jproteome.6b00049 27018767

[B16] Greco C. M. Berman R. F. Martin R. M. Tassone F. Schwartz P. H. Chang A. et al. (2006). Neuropathology of fragile X-associated tremor/ataxia syndrome (FXTAS). *Brain* 129(Pt 1), 243–255. 10.1093/brain/awh683 16332642

[B17] Hagerman R. J. Hagerman P. (2016). Fragile X-associated tremor/ataxia syndrome - features, mechanisms and management. *Nat. Rev. Neurol*. 12 403–412. 10.1038/nrneurol.2016.82 27340021

[B18] Hagerman R. Hagerman P. (2021). Fragile X-associated tremor/ataxia syndrome: pathophysiology and management. *Curr. Opin. Neurol*. 34 541–546. 10.1097/WCO.0000000000000954 33990099 PMC8412174

[B19] Hall D. A. Birch R. C. Anheim M. Jønch A. E. Pintado E. O’Keefe J. et al. (2014). Emerging topics in FXTAS. *J. Neurodev. Disord*. 6:31. 10.1186/1866-1955-6-31 25642984 PMC4141265

[B20] Hasin Y. Seldin M. Lusis A. (2017). Multi-omics approaches to disease. *Genome Biol*. 18:83. 10.1186/s13059-017-1215-1 28476144 PMC5418815

[B21] Havelund J. F. Heegaard N. H. H. Færgeman N. J. K. Gramsbergen J. B. (2017). Biomarker research in Parkinson’s Disease using metabolite profiling. *Metabolites* 7:42. 10.3390/metabo7030042 28800113 PMC5618327

[B22] Holm K. N. Herren A. W. Taylor S. L. Randol J. L. Kim K. Espinal G. et al. (2021). Human cerebral cortex proteome of Fragile X-associated tremor/ataxia syndrome. *Front. Mol. Biosci*. 7:600840. 10.3389/fmolb.2020.600840 33585555 PMC7879451

[B23] Jacquemont S. Farzin F. Hall D. Leehey M. Tassone F. Gane L. et al. (2004). Aging in individuals with the FMR1 mutation. *Am. J. Ment. Retard.* 109 154–164. 10.1352/0895-80172004109<154:AIIWTF<2.0.CO;2 15000674 PMC3249442

[B24] Jacquemont S. Hagerman R. J. Leehey M. Grigsby J. Zhang L. Brunberg J. A. et al. (2003). Fragile X premutation tremor/ataxia syndrome: molecular, clinical, and neuroimaging correlates. *Am. J. Hum. Genet*. 72 869–878. 10.1086/374321 12638084 PMC1180350

[B25] Jin P. Duan R. Qurashi A. Qin Y. Tian D. Rosser T. C. et al. (2007). Pur alpha binds to rCGG repeats and modulates repeat-mediated neurodegeneration in a Drosophila model of fragile X tremor/ataxia syndrome. *Neuron* 55 556–564. 10.1016/j.neuron.2007.07.020 17698009 PMC1994817

[B26] Johnson E. C. B. Dammer E. B. Duong D. M. Ping L. Zhou M. Yin L. et al. (2020). Large-scale proteomic analysis of Alzheimer’s disease brain and cerebrospinal fluid reveals early changes in energy metabolism associated with microglia and astrocyte activation. *Nat. Med*. 26 769–780. 10.1038/s41591-020-0815-6 32284590 PMC7405761

[B27] Joilin G. Leigh P. N. Newbury S. F. Hafezparast M. (2019). An overview of MicroRNAs as biomarkers of ALS. *Front. Neurol*. 10:186. 10.3389/fneur.2019.00186 30899244 PMC6416171

[B28] Kao Y. C. Ho P. C. Tu Y. K. Jou I. M. Tsai K. J. (2020). Lipids and Alzheimer’s Disease. *Int. J. Mol. Sci*. 21:1505. 10.3390/ijms21041505 32098382 PMC7073164

[B29] Kodam P. Sai Swaroop R. Pradhan S. S. Sivaramakrishnan V. Vadrevu R. (2023). Integrated multi-omics analysis of Alzheimer’s disease shows molecular signatures associated with disease progression and potential therapeutic targets. *Sci. Rep*. 13:3695. 10.1038/s41598-023-30892-6 36879094 PMC9986671

[B30] Kong H. E. Lim J. Zhang F. Huang L. Gu Y. Nelson D. L. et al. (2019). Metabolic pathways modulate the neuronal toxicity associated with fragile X-associated tremor/ataxia syndrome. *Hum. Mol. Genet*. 28 980–991. 10.1093/hmg/ddy410 30476102 PMC6400045

[B31] Langfelder P. Cantle J. P. Chatzopoulou D. Wang N. Gao F. Al-Ramahi I. et al. (2016). Integrated genomics and proteomics define huntingtin CAG length-dependent networks in mice. *Nat. Neurosci*. 19 623–633. 10.1038/nn.4256 26900923 PMC5984042

[B32] Leehey M. A. Berry-Kravis E. Goetz C. G. Zhang L. Hall D. A. Li L. et al. (2008). FMR1 CGG repeat length predicts motor dysfunction in premutation carriers. *Neurology* 70(16 Pt 2), 1397–1402. 10.1212/01.wnl.0000281692.98200.f5 18057320 PMC2685188

[B33] Lin C. N. Huang C. C. Huang K. L. Lin K. J. Yen T. C. Kuo H. C. (2019). A metabolomic approach to identifying biomarkers in blood of Alzheimer’s disease. *Ann. Clin. Transl. Neurol*. 6 537–545. 10.1002/acn3.726 30911577 PMC6414491

[B34] Louis E. Moskowitz C. Friez M. Amaya M. Vonsattel J. P. (2006). Parkinsonism, dysautonomia, and intranuclear inclusions in a fragile X carrier: a clinical-pathological study. *Mov. Disord*. 21 420–425. 10.1002/mds.20753 16250026

[B35] Menden K. Francescatto M. Nyima T. Blauwendraat C. Dhingra A. Castillo-Lizardo M. et al. (2023). A multi-omics dataset for the analysis of frontotemporal dementia genetic subtypes. *Sci. Data* 10:849. 10.1038/s41597-023-02598-x 38040703 PMC10692098

[B36] Nahalkova J. Volkmann I. Aoki M. Winblad B. Bogdanovic N. Tjernberg L. O. et al. (2010). CD147, a gamma-secretase associated protein is upregulated in Alzheimer’s disease brain and its cellular trafficking is affected by presenilin-2. *Neurochem. Int*. 56 67–76. 10.1016/j.neuint.2009.09.003 19751784

[B37] Napoli E. Song G. Schneider A. Hagerman R. Eldeeb M. A. Azarang A. et al. (2016). Warburg effect linked to cognitive-executive deficits in FMR1 premutation. *FASEB J*. 30 3334–3351. 10.1096/fj.201600315R 27335370 PMC5024697

[B38] Reddy J. S. Heath L. Linden A. V. Allen M. Lopes K. P. Seifar F. et al. (2024). Bridging the gap: multi-omics profiling of brain tissue in Alzheimer’s disease and older controls in multi-ethnic populations. *Alzheimers Dement*. 20 7174–7192. 10.1002/alz.14208 39215503 PMC11485084

[B39] Rodriguez-Revenga L. Madrigal I. Pagonabarraga J. Xunclà M. Badenas C. Kulisevsky J. et al. (2009). Penetrance of FMR1 premutation associated pathologies in fragile X syndrome families. *Eur. J. Hum. Genet*. 17 1359–1362. 10.1038/ejhg.2009.51 19367323 PMC2986640

[B40] Salcedo-Arellano M. J. Johnson M. D. McLennan Y. A. Hwang Y. H. Juarez P. McBride E. L. et al. (2023). Brain metabolomics in Fragile X-associated tremor/ataxia syndrome (FXTAS). *Cells* 12:2132. 10.3390/cells12172132 37681866 PMC10487256

[B41] Schilder B. M. Navarro E. Raj T. (2022). Multi-omic insights into Parkinson’s Disease: from genetic associations to functional mechanisms. *Neurobiol. Dis*. 163:105580. 10.1016/j.nbd.2021.105580 34871738 PMC10101343

[B42] Sellier C. Rau F. Liu Y. Tassone F. Hukema R. K. Gattoni R. et al. (2010). Sam68 sequestration and partial loss of function are associated with splicing alterations in FXTAS patients. *EMBO J*. 29 1248–1261. 10.1038/emboj.2010.21 20186122 PMC2857464

[B43] Shao Y. Le W. (2019). Recent advances and perspectives of metabolomics-based investigations in Parkinson’s disease. *Mol. Neurodegener*. 14:3. 10.1186/s13024-018-0304-2 30634989 PMC6330496

[B44] Skene N. G. Bryois J. Bakken T. E. Breen G. Crowley J. J. Gaspar H. A. et al. (2018). Genetic identification of brain cell types underlying schizophrenia. *Nat. Genet*. 50 825–833. 10.1038/s41588-018-0129-5 29785013 PMC6477180

[B45] Song G. Napoli E. Wong S. Hagerman R. Liu S. Tassone F. et al. (2016). Altered redox mitochondrial biology in the neurodegenerative disorder fragile X-tremor/ataxia syndrome: use of antioxidants in precision medicine. *Mol. Med*. 22 548–559. 10.2119/molmed.2016.00122 27385396 PMC5082295

[B46] Subramanian I. Verma S. Kumar S. Jere A. Anamika K. (2020). Multi-omics data integration, interpretation, and its application. *Bioinform. Biol. Insights* 14:1177932219899051. 10.1177/1177932219899051 32076369 PMC7003173

[B47] Swarup V. Hinz F. I. Rexach J. E. Noguchi K. I. Toyoshiba H. Oda A. et al. (2019). Identification of evolutionarily conserved gene networks mediating neurodegenerative dementia. *Nat. Med*. 25 152–164. 10.1038/s41591-018-0223-3 30510257 PMC6602064

[B48] Tassone F. Greco C. M. Hunsaker M. R. Seritan A. L. Berman R. F. Gane L. W. et al. (2012). Neuropathological, clinical and molecular pathology in female fragile X premutation carriers with and without FXTAS. *Genes Brain Behav*. 11 577–585. 10.1111/j.1601-183X.2012.00779.x 22463693 PMC3965773

[B49] Tassone F. Hagerman R. J. Taylor A. K. Gane L. W. Godfrey T. E. Hagerman P. J. (2000). Elevated levels of FMR1 mRNA in carrier males: a new mechanism of involvement in the fragile-X syndrome. *Am. J. Hum. Genet*. 66 6–15. 10.1086/302720 10631132 PMC1288349

[B50] Tassone F. Pan R. Amiri K. Taylor A. K. Hagerman P. J. (2008). A rapid polymerase chain reaction-based screening method for identification of all expanded alleles of the fragile X (FMR1) gene in newborn and high-risk populations. *J. Mol Diagn*. 10 43–49. 10.2353/jmoldx.2008.070073 18165273 PMC2175542

[B51] Tassone F. Protic D. Allen E. G. Archibald A. D. Baud A. Brown T. W. et al. (2023). Insight and recommendations for fragile X-premutation-associated conditions from the fifth international conference on FMR1 premutation. *Cells* 12:2330. 10.3390/cells12182330 37759552 PMC10529056

[B52] Todd P. K. Paulson H. L. (2010). RNA-mediated neurodegeneration in repeat expansion disorders. *Ann. Neurol*. 67 291–300. 10.1002/ana.21948 20373340 PMC2852186

[B53] Tolosa E. Vila M. Klein C. Rascol O. (2020). LRRK2 in Parkinson disease: challenges of clinical trials. *Nat. Rev. Neurol*. 16 97–107. 10.1038/s41582-019-0301-2 31980808

[B54] Umoh M. E. Dammer E. B. Dai J. Duong D. M. Lah J. J. Levey A. I. et al. (2018). A proteomic network approach across the ALS-FTD disease spectrum resolves clinical phenotypes and genetic vulnerability in human brain. *EMBO Mol. Med*. 10 48–62. 10.15252/emmm.201708202 29191947 PMC5760858

[B55] Viode A. van Zalm P. Smolen K. K. Fatou B. Stevenson D. Jha M. et al. (2023). A simple, time- and cost-effective, high-throughput depletion strategy for deep plasma proteomics. *Sci. Adv*. 9:eadf9717. 10.1126/sciadv.adf9717 36989362 PMC10058233

[B56] Wertz M. H. Mitchem M. R. Pineda S. S. Hachigian L. J. Lee H. Lau V. et al. (2020). Genome-wide In Vivo CNS screening identifies genes that modify CNS neuronal survival and mHTT toxicity. *Neuron* 106 76–89.e8. 10.1016/j.neuron.2020.01.004. 32004439 PMC7181458

[B57] Yao P. J. Manolopoulos A. Eren E. Rivera S. M. Hessl D. R. Hagerman R. et al. (2024). Mitochondrial dysfunction in brain tissues and extracellular vesicles fragile X-associated tremor/ataxia syndrome. *Ann. Clin. Transl. Neurol*. 11 1420–1429. 10.1002/acn3.52040 38717724 PMC11187838

[B58] Zafarullah M. Durbin-Johnson B. Fourie E. S. Hessl D. R. Rivera S. M. Tassone F. (2021). Metabolomic biomarkers are associated with area of the pons in fragile X premutation carriers at risk for developing FXTAS. *Front. Psychiatry* 12:691717. 10.3389/fpsyt.2021.691717 34483988 PMC8415564

[B59] Zafarullah M. Li J. Salemi M. R. Phinney B. S. Durbin-Johnson B. P. Hagerman R. et al. (2023). Blood proteome profiling reveals biomarkers and pathway alterations in fragile X PM at risk for developing FXTAS. *Int. J. Mol. Sci*. 24:13477. 10.3390/ijms241713477 37686279 PMC10488017

[B60] Zafarullah M. Palczewski G. Rivera S. M. Hessl D. R. Tassone F. (2020). Metabolic profiling reveals dysregulated lipid metabolism and potential biomarkers associated with the development and progression of Fragile X-Associated Tremor/Ataxia Syndrome (FXTAS). *FASEB J*. 34 16676–16692. 10.1096/fj.202001880R 33131090 PMC7756608

[B61] Zhu X. Song Z. Zhang S. Nanda A. Li G. (2014). CD147: a novel modulator of inflammatory and immune disorders. *Curr. Med. Chem*. 21 2138–2145. 10.2174/0929867321666131227163352 24372217

