# Supplementary figures and images for: Integrated multi-omics profiling reveals novel molecular biomarkers and pathways associated with Fragile X-associated tremor/ataxia syndrome
Source: Front Mol Neurosci. 2026 Apr 17;19:1752903. doi: 10.3389/fnmol.2026.1752903 (PMC13132834; doi:10.3389/fnmol.2026.1752903)

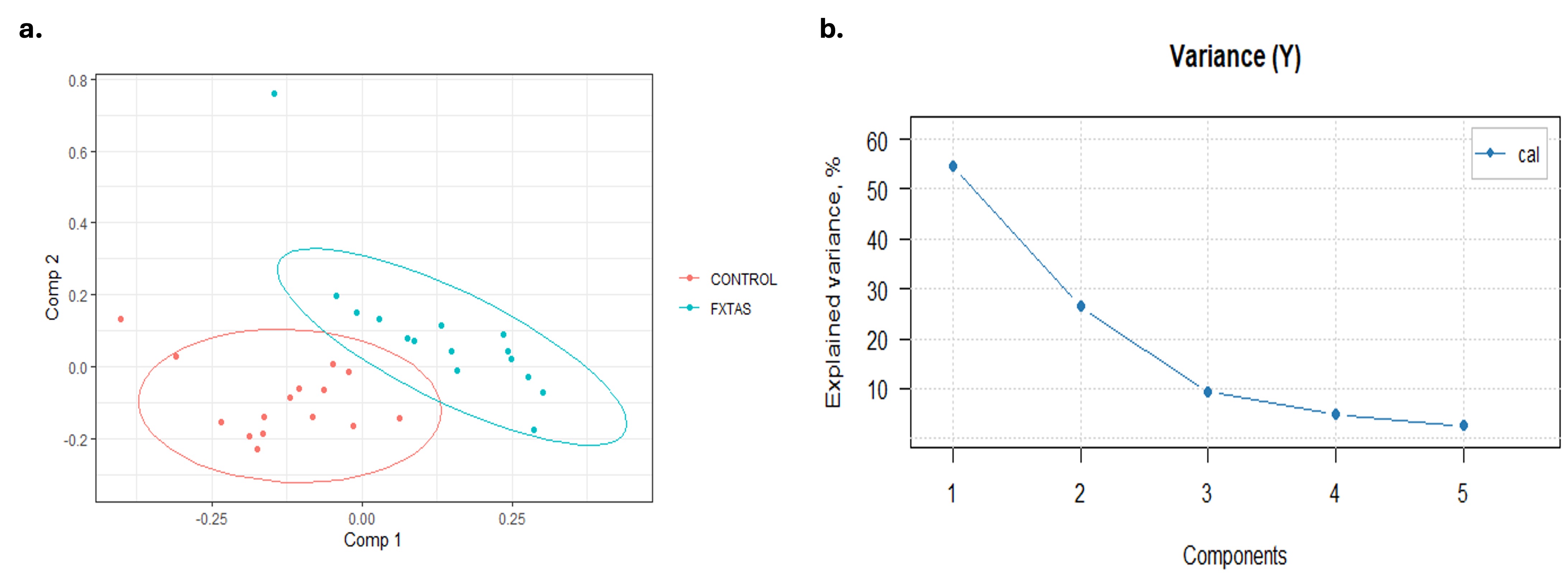

Supplement: Supplementary Figure 1 — The healthy control and Fragile X-associated tremor/ataxia syndrome (FXTAS) groups show high level of segregation. (a) Scatter plot showing the separation between control and FXTAS samples. (b) Screen plot showing the proportion of variance around 81.06% between both groups. [file Image_1.jpeg]

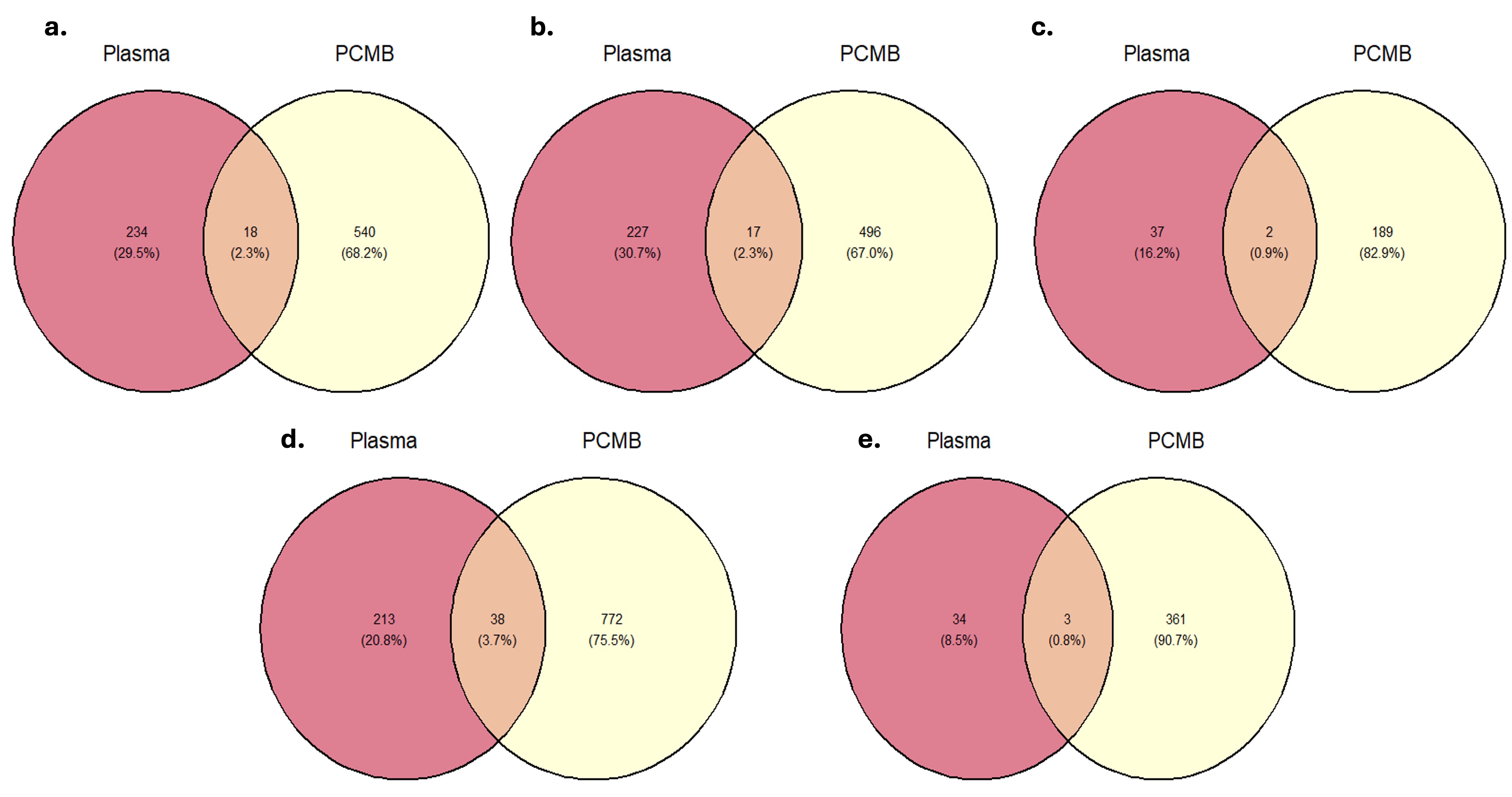

Supplement: Supplementary Figure 2 — Differentially expressed common proteins identified from PBMC and plasma profiling of healthy control (HC) and Fragile X-associated tremor/ataxia syndrome (FXTAS) patients. Venn diagram of proteins (a) significantly differentially expressed (P < 0.05). (b) Significantly associated with FXTAS stage at p < 0.05 in all samples. (c) Significantly associated with FXTAS stage at p < 0.05 in FXTAS patients. (d) Significantly associated with CGG repeat number at p < 0.05 in all samples. (e) Significantly associated with CGG repeat number at p < 0.05 in FXTAS patients. [file Image_2.jpeg]
